# Longitudinal study on steroid hormone variations during the second trimester of gestation: a useful tool to confirm adequate foetal development

**DOI:** 10.1186/s12884-021-03617-8

**Published:** 2021-02-09

**Authors:** Silvia Alonso, Sara Caceres, Daniel Vélez, Luis Sanz, Gema Silvan, Maria Jose Illera, Juan Carlos Illera

**Affiliations:** 1 Departamento de Fisiología, Facultad de Veterinaria, Universidad Complutense de Madrid, 28040 Madrid, Spain; 2 Department of Statistics and Operational Research, Faculty of Mathematics, University Complutense of Madrid, 28040 Madrid, Spain

**Keywords:** Cortisol, Estriol, Estrone sulphate, Progesterone, Pregnancy, Foetal development

## Abstract

**Background:**

The interaction of hormonal factors are crucial for good foetal development. During the second trimester of gestation, most of the main physiological processes of foetal development occur. Therefore, the aim of this study was to determine the variations in the physiological levels of cortisol, estriol, estrone sulphate, and progesterone during the second trimester (weeks 12–26) in order to establish normal ranges that can serve as indicators of foetal well-being and good functioning of the foetal-placental unit.

**Methods:**

Saliva samples from 106 pregnant women were collected weekly (from week 12 to week 26 of gestation), and hormonal measurements were assayed by an enzyme immunoassay. The technique used for hormone measurements was highly sensitive and served as a non-invasive method for sample collection.

**Results:**

The results revealed a statistically significant (*p*<0.05) difference between cortisol, progesterone, and oestrogens throughout the second trimester, with a more substantial relationship between oestrogens and progesterone [P4-E3 (r=0.427); P4-E1SO4 (r=0.419)]. By analysing these hormone concentrations, statistically significant (*p*<0.05) elevations in progesterone, cortisol, and estriol levels were found at the 16^th^ [(P4 (0.78±0.088), C(1.99±0.116), E3(2.513±0.114)]; 18th [(P4 (1.116±0.144), C(3.409±0.137), E3(3.043±0.123)] and 23rd week of gestation [(P4(1.36±0.153), C(1.936±0.11), E3(2.657±0.07)]. Estrone sulphate levels appeared to increase progressively throughout the second trimester [from 1.103±0.03 to 2.244±0.09].

**Conclusion:**

The 18th week of gestation seems to constitute a very important week during foetal adrenal development, and the analysis of the main hormones involved in foetal development, provided more precise information regarding the proper functioning of the foetal unit and foetal development.

## Background

The physiological processes involved in pregnancy mainly depend on the interactions of hormonal factors, where the foetal–maternal endocrine system plays an important role. The placenta modulates the regulation of distinct factors by feedback mechanisms for correct foetal development [1]. The placenta and foetal adrenal gland are the organs responsible for producing large amounts of steroid hormones in the second and third trimester of gestation [2]. Progestogens, oestrogens, androgens, and glucocorticoids are secreted at different times during gestation, and their interactions modulate different cellular and physiological responses [1].

Most oestrogens are produced by the placenta and are sulphated or inactivated in the foetal liver to protect the foetus from excess oestrogen levels. Foetal exposure to abnormal concentrations of steroid hormones can have a negative impact on foetal development [3].

The main steroid hormones produced in the foetal adrenal glands are pregnenolone (P5), dehydroepiandrosterone (DHEA), and cortisol (C). P5 and DHEA act as substrates for the synthesis of progesterone (P4) and oestrogens in the placenta [2].

P4 and oestrogens are progressively secreted throughout gestation, and P4 levels are responsible for the maintenance of pregnancy [1]. Oestrogens, such as estriol (E3) and estrone sulphate (E1SO4), are produced by the placenta from dehydroepiandrosterone sulphate (DHEA-S), which is synthesised exclusively by the foetal adrenal glands [4]. E1SO4 acts as a reserve for the peripheral formation of bioactive estrogenic forms [5]. Likewise, E3 is the dominant oestrogen hormone during pregnancy and is used as an index of the state of the foetal-placental unit since its production depends on the synthesis capacity of the foetus and placenta together [4]. C levels are associated with the placental synthesis of crucial oestrogens for the maintenance of pregnancy, [5] playing an important role throughout pregnancy, and are responsible for maintaining intrauterine homeostasis.

Immunoassays of maternal blood samples are used for steroid concentration during pregnancy [6]. However, new methods using amniotic fluid samples, such as liquid chromatography-tandem mass spectrometry (LC-MS/MS), have been given consideration since they are more reliable and sensitive [7]. In this study, we present the use of saliva samples for the determination of steroid hormone levels with immunoassay techniques as sensitive and non-invasive methods.

The knowledge of the interactions of the main steroid hormones during the second trimester of gestation is poor. Considering that significant foetal development events occur during the second trimester of pregnancy, [8] the aim of this study was to determine the levels of P4, E3, E1SO4, and C in saliva samples of pregnant women during the second trimester of pregnancy (from weeks 12–26) in order to evaluate hormonal variations and their interactions.

## Methods

### Participant recruitment

The study was carried out at the Nuevo Belén Clinical University Hospital (Madrid, Spain) in collaboration with the Department of Animal Physiology of the University Complutense of Madrid (Spain). The process of patient recruitment and sample collection were carried out for over a year (Sept 2017- Sept 2018). An informed consent was obtained from all participants recruited on this study.

A total of 161 healthy pregnant women without any pregnancy complications, aged between 27 and 44 years (34.88 ± 3.29) were recruited (data from all recruited woman are summarized in Table 1). Two of the initial participants dropped out before the beginning of the study, which made us commence with a total of 159 women, of whom they dropped out throughout the study reaching a total of 106 who completed the study. All women who fulfilled the inclusion criteria were included during the recruitment period. The criteria for inclusion were good overall health, age > 18 years, no current systemic pharmacotherapy, and no smoking. They were also required to be in the second trimester. The exclusion criteria were the following: kidney disease, thyroid disease, autoimmune disease, cancer, pregestational diabetes, gestational diabetes, pregestational hypertension, overweight, obesity, in vitro fertilisation with heparin treatment, and current steroid treatment.

**Table 1:** Data from all recruited women

	All participants recruited
	*N*=159
	Participants that completed the study
	*N*=106 (Dropout rate=33.3%)
Mean age (years)	34.88±3.29
Pre-pregnancy body mass index (BMI) (n, %)	
Normal weight	97 (91.5%)
Underweight	9 (8.4 %)
Parity (n, %)	
Primiparous	66 (62.2%)
Multiparous	40 (37.7%)
Current pregnancy	
Mean gestational age	38 weeks + 6 days
Birth at 37 weeks	17 (16%)
Birth at 38 weeks	30 (28.3%)
Birth at 39 weeks	24 (22.6%)
Birth at 40 weeks	21 (19.8%)
Birth at 41 weeks	16 (15%)
Mode of delivery	
Spontaneous birth	63 (59.4%)
Induction of labour (postdates + prolonged rupture of membranes + other reasons)	43 (40.5%)
Induction of labour for postdates (>41+4)	6 (6.3%)
Vaginal birth	83 (78.3%)
Caesarean delivery without trial of labour	9 (8.4%)
Caesarean delivery after trial of labour	14 (13.2%)
Rupture of membranes	
>24 hours	31 (29.2%)
<24 hours	75 (70.8%)
Newborn	
Mean birth weight (g)	3479±356
Apgar score <7	
1 min	8 (7.5%)
5 min	4 (3.7%)
10 min	2 (1.8%)

### Saliva sample collection and hormone measurements

Saliva samples were collected weekly from eligible women during the second trimester of pregnancy (from weeks 12–26) with a Salivette collection tube. All samples were collected at an established time (10:00 am ± 1 hour). The Salivette tube was centrifuged for 15 min at 2000 ×g and 4°C. The obtained saliva was stored at a temperature of − 20°C until further hormonal analysis.

P4 (ab: C914), E1SO4 (ab: R522-2), E3 (ab: 4835), and C (ab: R4866) concentrations were assayed by enzyme immunoassay (EIA). All hormone concentrations are expressed in ng/ml.

The validation technique parameters (recovery rates, sensitivity, intra- and inter-assay coefficients of variation, and parallelism) were assayed as previously reported by Illera et al., (2014) [9]. The EIA techniques and the antibodies used were developed and validated in the Endocrinology Laboratory of the Department of Animal Physiology (Faculty of Veterinary Medicine, Universidad Complutense de Madrid).

### Statistical analysis

The hormonal concentration data were analysed with the SAS 9.4 program. Descriptive statistical analysis for mean and standard deviation of each hormone based on the week of delivery was performed,, Repeated measures ANOVA was perfomed in order to compare the evolution of P4, E1SO4, E3, and C concentrations throughout the addressed experimental weeks. Repeated measures correlation coefficient (rmcorr) between hormonal values during the third trimester were estimated using PROC MIXED procedures by SAS.

Validation technique parameters (recovery rates, sensitivity, and intra- and inter-assay coefficients of variation) were calculated as previously described by Andreasson et al., 2015 [10]. Parallelism was calculated using ANOVA analysis [11]. Data are expressed as the mean ± standard error. In all statistical comparisons, *p*-values of *p* < 0.05 were considered statistically significant.

## Results

### Clinical characteristics of the study group

A total of 159 healthy pregnant women were recruited at the obstetric clinic and prospectively followed from week 12 of gestation to delivery (Table 1). Of those, 106 (66,6 %) completed the study. From all women who completed the study, the mean age of the mother was 34.88±3.29 years. Most of the women had a pre-pregnancy normal weight (91,5 %) while 8.4 % had a pre-pregnancy underweight. 62 % of the women were primiparous and 37.7 % were multiparous. The mean gestational age was 38±6 weeks. The mean birth weight was 3479± 356 g.

### Validation parameters

The sensitivity of the EIA technique was verified by a low limit of detection and was calculated in 10 consecutive assays. Results from low limit detection were: P4 = 12.81 pg/well, C = 2.48 pg /well, E3 = 1.87 pg/well and E1SO4 = 4.24 pg/well. The assessment of the recovery rates of the conjugate gave the following results: P4, 0.9:1; C, 1.3:1; E3, 1.2:1; and E1SO4, 1.2:1 moles. The recovery of the enzyme activity after conjugation was more than 85 % in all cases. The precision of P4, C, E3, and E1SO4 EIAs was determined by calculating the intra- and inter-assay coefficients of variation (CV%). Results from CV% were: P4 = Intra: 3.6 ± 0.86 %, Inter: 5.2 ± 0.91 %; C = Intra: 4.9 ± 0.92 %, Inter: 6.9 ± 1.13 %; E3 = Intra: 7.1 ± 1.16 %, Inter: 8.6 ± 1.76 %; and E1SO4 = Intra: 7.8 ± 1.14 %, Inter: 9.4 ± 2.16 %. In order to determine the effects of saliva on the standard curve, the standard curves with saliva samples were run in parallel with the standard dose–response curve. Correlations between both standards curves resulted in a good degree of parallelism between both standard curves for the hormones studied (P4, R^2^ = 0.86; C, R^2^ = 0.84; E3, R^2^ = 0.89; and E1SO4, R^2^ = 0.83).

### Hormonal concentrations

**Fig. 1:**
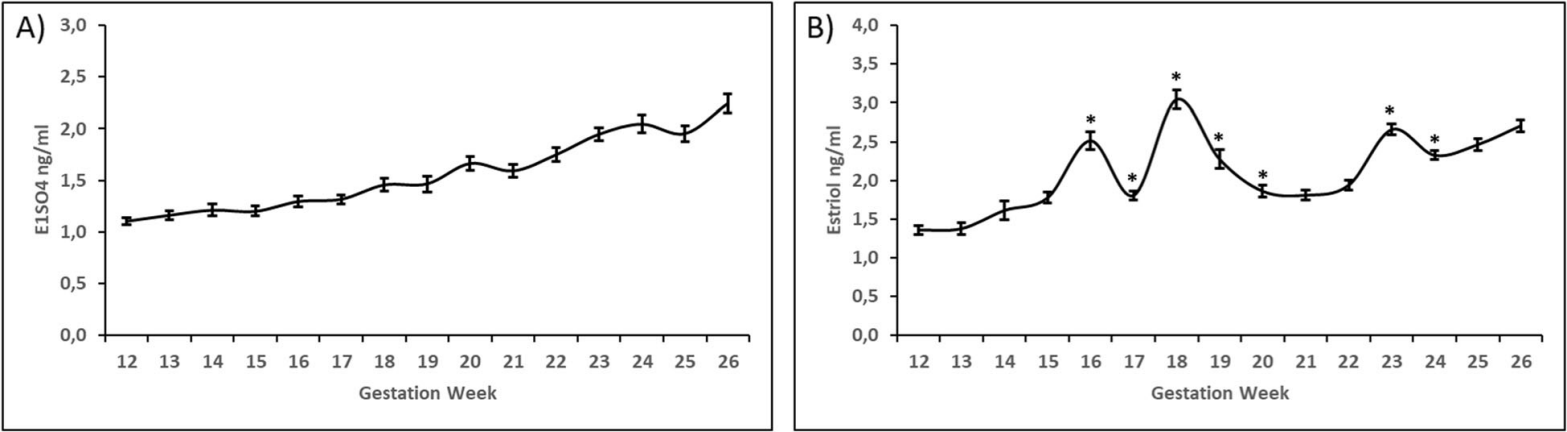
Estrone sulphate (**a**) and estriol (**b**) concentrations during second of pregnancy (12–26 weeks). Values are represented by means ± SD. * denoted statistical significance throughout experimental weeks

A progressive increase in E1SO4 and E3 levels (Fig. 1a, b) was shown throughout the second trimester of gestation (weeks 12–26) (E1SO4: 1.104 ± 0.032 to 2.245 ± 0.093; E3: 1.358 ± 0.059 to 2.708 ± 0.074). Increases in E3 levels (*p* < 0.05) were also found during the 16th, 18th, and 23rd weeks, with the greatest increase found during the 18th week (3.044 ± 0.123).

**Fig. 2:**
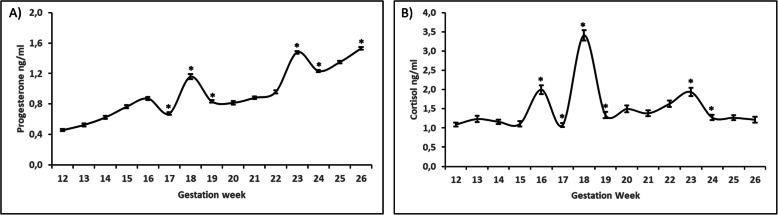
Progesterone (**a**) and cortisol (**b**) concentrations during second of pregnancy (12–26 weeks). Values are represented by means ± SD. * denoted statistical significance throughout experimental weeks

The same progressive increase in P4 levels was found (Fig. 2a) during the second trimester (0.456 ± 0.014 to 1.530 ± 0.018), and two clinically significant elevations in the 18th and 23rd weeks were found. Nevertheless, a slight increase in P4 levels was found in the 16th week, but there was not a statistically significant increase in E3 concentrations.

Although a progressive increase in C levels during the weeks studied was not found (Fig. 2b), significant increases in C levels were shown in the 16th, 18th, and 23rd weeks, with the greatest increase during the 18th week (3.409 ± 0.138).

Being rmcorr the correlation taking into account repeated measures, positive correlations were found between the hormones studied (Table 2), with stronger correlations between P4 and E3 (rmcorr = 0.407) and P4 and E1SO4 (rmcorr = 0.443) and lower correlations between C and E3 (rmcorr = 0.257) and C and P4 (rmcorr = 0.147).

**Table 2:** Repeated measures correlation (rmcorr) of P4, C, E3, and E1SO4 concentrations in the second trimester of gestation

From week 12 to 26		
$v1$	$v2$	${\rho }_{v1v2}$
CORTISOL	ESTRIOL	0.2570254
CORTISOL	PROGEST	0.30937017
CORTISOL	E1SO4	0.0921688
ESTRIOL	PROGEST	0.407928
ESTRIOL	E1SO4	0.14707268
PROGESTE	E1SO4	0.44360565

## Discussion

Understanding of the interactions of hormonal factors displayed during the second trimester of gestation is crucial. It is difficult to directly compare these results with other previous studies due to the different methodologies used, such as hormonal measurements at specific moments during pregnancy (especially during the last weeks of pregnancy) or measurements using different biological samples. Some authors have suggested the need for longitudinal studies to explain the different types of prenatal stress and their relationship with maternal physiology and the outcomes of childbirth [12].

The main contribution of this study is that it shows the physiological variations of four of the main hormones involved during the second trimester of pregnancy (C, E3, P4, and E1SO4). However, more longitudinal studies of women during the second trimester are needed to address these findings. We also incorporated the analysis of these hormones in saliva samples, a non-invasive method for measure good-functioning of foetal-unit development. Also, the technique used in this study, developed by Illera et al., (2014) [9] was found to be highly sensitive and accurate. Therefore, we implemented the analysis of these hormones in saliva samples, a useful non-invasive rising method where the collection of the sample does not cause stress and does not require training of health personnel.

Our results revealed that the four hormones analysed are positively correlated, with the most important correlation between oestrogens (E3 and E1SO4) and P4 in the second trimester of gestation. Indeed, greater increases in C, E3, and P4 levels were found in the 16th, 18th, and 23rd weeks of gestations.

Defining the exact moment during gestation in which the foetal adrenal cortex begins to function, as well as defining the mechanism by which it begins to function, is complex [13]. Howland et al. [14] suggested that the foetal HHA axis starts to function autonomously at around midgestation, reaching full development at 2 years of age. Nonetheless, some authors have stated that the foetal adrenal gland functions autonomously starting during the 8th week of gestation [2].

Based on our results, the increase in C found during the 18th week of gestation can be correlated with the autonomous function of the adrenal gland, which leads to higher secretion of P4 and higher oestrogen levels. However, other authors stated that the synthesis of de novo C begins in the definitive zone of the adrenal gland at week 23 of gestation [15].

It is known that the second trimester of pregnancy is the most sensitive to variations of maternal C concentrations could causing adverse effects on foetal development [16]. There is a controversy regarding C levels and its association with stress. Several studies correlated C levels with maternal stress, [17] whereas other authors suggested that C variations are affected by biological factors rather than lifestyle factors as stress [18, 19]. In accordance with these authors, our results did not revealed any association with C levels and maternal stress.

C levels has been also associated with birth weight [18] or even children weight (2 to 16 years old) [16]. As some authors determine that higher C levels during pregnancy may be a risk factor for low birth weight, [18] other authors suggested that higher C levels are associated with children overweight [16].

Some authors revealed that the combined effect of high levels of estradiol and P4 affected to the production of coagulation factor and, therefore, develop a prothrombotic state during first trimester of pregnancy [20]. However, the increased in E3 and P4 levels in this study cannot be correlated with this phenomenon, as all pregnant women did not have any complications during pregnancy.

Foetal movements are considered a sign of maturity of the neurological system and of the well-being of the foetus [21]. A mother’s perception of these movements begins between weeks 16 and 20 of pregnancy [22]. As early as week 24, the foetus is capable to integrate the exteroceptive and interoceptive stimuli, such as feelings, memories, and emotions, [23, 24] being able at this stage to integrate external sensory information and to develop a physiological and behavioural response [24]. At week 16, the foetus is also able to move in a coordinated manner. These movements can be perceived by the mother and detected by ultrasound techniques, [24] and the increase in the intensity of these movements have been associated with higher C levels, [25] which can be related with the increase in C levels found at 16th week.

It is also during these weeks that the development of the sensorimotor capacity of the foetus begins. At around week 16, the foetus develop spontaneous motor activity during active sleep, [26] and nociceptive reactions can be recorded starting at week 20 [27]. At around 22–23 week fetus develop the capacity to detect and memorize the consequences of spontaneous activity as arm movements towards mouth and eyelids [26].

The strengths of our study included the comparison of the four hormones simultaneously during the same gestational period and their analysis in saliva. However, several limitation has been noted. Firstly, it was the participant recruitment as well as the significant drop-out rate. Secondly, another limitation found was the lack of continuity in the weekly collection of samples by the participants due to forgetfulness.

Despite the positive correlations found between the courses of different hormone levels, it can’t be said firmly whether these positive correlations are due to interactions of the hormones studied or whether they independently rise over time. However, knowing the hormonal variations throughout the second trimester of pregnancy could serve as an indicator of proper functioning of the adrenal axis, leading to normal foetal development.

## Conclusions

The knowledge and analysis of the factors that influence foetal development and their possible consequences can help to improve the preventative measures provided by health professionals during pregnancy.

The increase in C, P4, and E3 levels at the 18th week of gestation can be related to the autonomous function of the foetal adrenal gland, suggesting that is a key week for foetal development. Although saliva based diagnostics still require further investigation for its implementation as analytical method, the analysis of the C, P4, and oestrogen concentrations in saliva samples during the second trimester of pregnancy (specifically at 18 th week), is a non-invasive and low-cost technique that can be a very useful tool to confirm the favourable development and functioning of the foetal endocrine system.

The results obtained in this exploratory study contribute to the generation of new hypotheses regarding the study of the development in the second trimester of gestation.

## Data Availability

The datasets generated and/or analysed during the current study are not publicly available due to the women who participated in the study did not agree to use their individual data in the signed consent document for participation in the research, but are available from the corresponding author on reasonable request.

## Abbreviations

C: Cortisol
E3: Estriol
E1SO4: Estrone sulphate
EIA: Enzyme immunoassay
P4: Progesterone
Rmcorr: Repeated measures correlation coefficient
